# The Role Of BMPs in the Regulation of Osteoclasts Resorption and Bone Remodeling: From Experimental Models to Clinical Applications

**DOI:** 10.3389/fimmu.2022.869422

**Published:** 2022-04-26

**Authors:** Tatjana Bordukalo-Nikšić, Vera Kufner, Slobodan Vukičević

**Affiliations:** Laboratory for Mineralized Tissues, Center for Translational and Clinical Research, University of Zagreb School of Medicine, Zagreb, Croatia

**Keywords:** BMPs, osteoclast differentiation, bone resorption, osteoblast-osteoclast coupling, bone fracture healing, recombinant BMP therapy

## Abstract

In response to mechanical forces and the aging process, bone in the adult skeleton is continuously remodeled by a process in which old and damaged bone is removed by bone-resorbing osteoclasts and subsequently is replaced by new bone by bone-forming cells, osteoblasts. During this essential process of bone remodeling, osteoclastic resorption is tightly coupled to osteoblastic bone formation. Bone-resorbing cells, multinuclear giant osteoclasts, derive from the monocyte/macrophage hematopoietic lineage and their differentiation is driven by distinct signaling molecules and transcription factors. Critical factors for this process are Macrophage Colony Stimulating Factor (M-CSF) and Receptor Activator Nuclear Factor-κB Ligand (RANKL). Besides their resorption activity, osteoclasts secrete coupling factors which promote recruitment of osteoblast precursors to the bone surface, regulating thus the whole process of bone remodeling. Bone morphogenetic proteins (BMPs), a family of multi-functional growth factors involved in numerous molecular and signaling pathways, have significant role in osteoblast-osteoclast communication and significantly impact bone remodeling. It is well known that BMPs help to maintain healthy bone by stimulating osteoblast mineralization, differentiation and survival. Recently, increasing evidence indicates that BMPs not only help in the anabolic part of bone remodeling process but also significantly influence bone catabolism. The deletion of the BMP receptor type 1A (BMPRIA) in osteoclasts increased osteoblastic bone formation, suggesting that BMPR1A signaling in osteoclasts regulates coupling to osteoblasts by reducing bone-formation activity during bone remodeling. The dual effect of BMPs on bone mineralization and resorption highlights the essential role of BMP signaling in bone homeostasis and they also appear to be involved in pathological processes in inflammatory disorders affecting bones and joints. Certain BMPs (BMP2 and -7) were approved for clinical use; however, increased bone resorption rather than formation were observed in clinical applications, suggesting the role BMPs have in osteoclast activation and subsequent osteolysis. Here, we summarize the current knowledge of BMP signaling in osteoclasts, its role in osteoclast resorption, bone remodeling, and osteoblast–osteoclast coupling. Furthermore, discussion of clinical application of recombinant BMP therapy is based on recent preclinical and clinical studies.

## Introduction

Bone homeostasis can be defined through balance of bone formation and bone resorption. In bone remodeling, a continuous and dynamic process that is going on throughout life, old or damaged mineralized bone is removed by bone-resorbing cells, osteoclasts, and is replaced by new bone matrix (osteoid) made by osteoblasts. Osteoid subsequently becomes fully mineralized bone tissue (1).

Bone morphogenetic proteins (BMPs) were first discovered in 1965 by Marshall Urist as endogenous factors which could induce ectopic bone formation (2). Subsequent research confirmed the role of BMPs in bone formation (3, 4). Today, it is known that all BMPs do not have the same effect and some of them do not induce ectopic bone formation, but for osteogenic BMPs, namely BMP2, -4, -5, -6, -7 and -9, bone-inducing properties *in vitro* and *in vivo* have been shown (5, 6). While most of earlier research was focused on effect of BMPs on bone forming cells, like osteo- and chondroprogenitors, increasing evidence indicates that BMPs effect osteoclasts as well, influencing also bone resorption and impacting thus the overall bone homeostasis. In this review, we focused on the role of BMPs on osteoclast differentiation and function and subsequently on bone resorption, as observed on *in vivo* and *in vitro* models.

## General Aspects of BMP Family

BMPs are secreted signaling molecules which belong to the large protein family consisting of more than 30 ligands, called transforming growth factor-β (TGFβ) superfamily (7, 8) and comprise an evolutionary conserved family of cytokines required for numerous developmental processes. Among the TGFβ superfamily members, the bone-formation activity is unique to BMPs (9); however, it was shown that BMPs have many other biological activities (10). Since their isolation as promotors of bone and cartilage formation, BMPs have been extensively studied and, besides their confirmed role in bone and cartilage, have been found to hold multiple functions in the embryonic development of other tissues and organ systems, including blood vessels, brain, liver, heart, lung, gut, limb, eye, teeth, or kidney. The role of BMP family members in development was confirmed when the deletion of some *Bmp* genes (including *Bmp*2 and *Bmp*4) and their receptors resulted in early embryonic lethality, at the beginning of the development of most gastrointestinal organs (11). Although most BMPs are expressed in a diversity of tissues during embryogenesis (12–14), the expression of some BMP members becomes limited to specific tissues after birth (15).

BMP family members have been identified in vertebrates and invertebrates. Among vertebrates, BMPs have highly conserved structures shared by the members of the TGFβ superfamily. Based on structural homology and known functions, the BMP family members can be further classified into several subgroups, including the BMP2/4 group, BMP5/6/7/8 group, BMP9/10 group, and BMP12/13/14 group. BMP-3, -4, -5, and -6 are highly expressed in lung, whereas BMP7 is mostly expressed in kidney of human embryos (12, 15) and of adult mice (16). Further, BMP4, -7, and -14 are important for proper reproductive tissue development and BMP2, -3, and -7 contribute to cartilage regeneration (17). *In vitro* experiments using pluripotent mesenchymal progenitor C3H10T1/2 cells and preosteoblastic C2C12 cells showed that BMP-2, -6, and -9 exhibit high ability to induce both early and late osteogenic markers as well as matrix mineralization, while most BMPs can effectively promote the terminal differentiation of committed osteoblastic precursors and osteoblasts (18, 19). In contrast to other BMPs, BMP3 has been proposed to act as an inhibitor of osteogenic BMPs and antagonizes the osteogenic activity of BMP-2, -4, -6, -7, and -9 (20), while deletion of *Bmp*3 gene results in increased skeletal bone volume (21).

Before being secreted into extracellular space where they become active, BMPs, as well as other TGFβ superfamily members, are first synthesized and folded as precursor proteins in the cytoplasm. BMP precursors form dimers that are subsequently cleaved by proteases during secretion of mature BMP into extracellular space (22). Mature BMPs are secreted as monomers which contain three intramolecular disulfide bonds, whereas fourth disulfide bond dimerizes with another BMP monomer, producing a biologically active dimer which activates corresponding BMP receptors (23, 24).

BMPs have been shown to be activated through reassembling with their prodomain in the process where antagonistic proteins and decoy receptors modulate BMP activity (25). In contrast, TGFβ proteins form a latency complex where TGFβ in inactive form as homodimer, bound by latency-associated peptide (LAP) and latent TGFβ binding protein (LTBP), forms large latent complex (LLC) (26). For its activation, the noncovalent bond between LAP and TGFβ has to be disrupted. Among many activators of TGFβ, a significant role belongs to BMP1. Although able to induce bone and cartilage, BMP1 is not part of the TGFβ superfamily of proteins. Instead, it possesses a metalloproteinase structure and acts as a procollagen C-proteinase which regulates collagen maturation (27, 28). In a process of TGFβ activation, BMP1 cleaves LTBP at two distinct sites enabling thus subsequent cleavage of LAP by other matrix metalloproteinases and liberation of active TGFβ which can then exert its biological functions (29). BMP1 appears to be not only the activator of TGFβ, but is also a significant regulator of its activity (30, 31).

Like other TGFβ superfamily members, BMPs induce their effects through two types of serine-threonine kinase transmembrane receptors, type I and type II receptors. Upon binding to the receptors, a heterotetrameric complex is formed, consisting of two dimers of type I and type II receptors (32). BMPs are able of binding to type I receptors in the absence of type II receptors but their binding affinity increases when both type I and type II receptors are present (33). Activated receptor complexes at the cell surface activate two main types of intracellular pathways, canonical (SMAD-dependent signaling pathway) or non-canonical (p38 mitogen-activated protein kinase, p38 MAPK) (34, 35). Canonical signaling pathway is highly conserved and involves three types of intracellular signal transducer SMAD molecules. Phosphorylated SMAD proteins form complex accumulating in the nucleus, where it binds to the responsive DNA elements and regulates target gene expression (36). On the other hand, non-canonical pathway includes activation of different pathways associated with various protein kinases, like Rho-GTPase, JNK/P38, PI3K/AKT, and MAPK pathway (37).

## Osteoclast Differentiation and *In Vitro* Models

**Figure 1:**
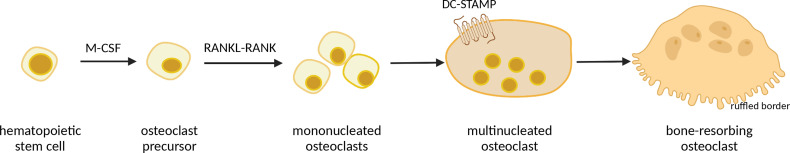
Schematic representation of osteoclast differentiation. Macrophage Colony Stimulating Factor (M-CSF) induces hematopoietic stem cells to become osteoclast precursors, which, under influence of Receptor-Activated Nuclear κB ligand (RANKL) develop into mononucleated osteoclasts. Further, mononucleated osteoclasts undergo fusion, mediated by DC-STAMP protein, into giant multinucleated osteoclasts, which then, upon interaction with osteoblasts, differentiate into mature bone-resorbing osteoclasts with ruffled border, which secrete acids and matrix metalloproteinases. Image created with BioRender.com.

Osteoclasts, cells primarily responsible for bone resorption, develop from hematopoietic stem cells in bone marrow, passing through several stages of differentiation (38). The two main differentiation factors that drive osteoclast maturation are Macrophage Colony Stimulating Factor (M-CSF) and Receptor-Activated Nuclear κB ligand (RANKL) (39–41), recognized by RANK (a RANKL receptor) expressed on osteoclast surface (42). During maturation, pre-osteoclasts differentiate into mononuclear cells expressing tartrate-resistant acid phosphatase (TRAP), and those TRAP-positive, mononuclear cells then fuse together into giant, multinucleated and polarized mature osteoclasts which can degrade skeletal matrix by secreting lytic enzymes (42, 43). The process of preosteoclast fusion is mediated by transmembrane protein DC-STAMP (44). Bone resorption occurs at the ruffled border, a morphological structure specific for mature osteoclasts consisting of complex folds of plasma membrane surrounded by an actin ring, adherently to the bone surface (45) (**Figure 1**). Osteoclast formation and subsequent bone resorption are inhibited by osteoprotegerin (OPG), a soluble factor produced by osteoblasts (41, 46).

Bone cell morphology, differentiation patterns and signal transduction are studied using widely used bone cell cultures as models *in vitro*. Single cell-type culture is commonly used, however, this model cannot reliably reproduce signal transduction between different cell types. On the other hand, simultaneous presence of osteoblasts and osteoclasts can mimic cellular cross-talk and mechanisms of intercellular communication (47). In preclinical studies, usage of co-culture of osteoblasts and osteoclasts is advantageous as it presents more relevant model of bone remodeling process (48).

When describing co-cultures of osteoblasts and osteoclasts, indirect or direct models can be utilized. In indirect models, use of conditioned media (media from one cell type transferred to the other) or transwell inserts, which provide two culture surfaces in the same well by the use of permeable insert, allow the exchange of soluble factors, but without a direct contact between two cell types. Direct co-cultures imply both cell types on the same surface, in two-dimensional (2D) cell culture, or in a three-dimensional scaffold, which allows the immediate physical contact between cell types and enables exploring the effects of membrane-bound signaling factors (49).

**Figure 2:**
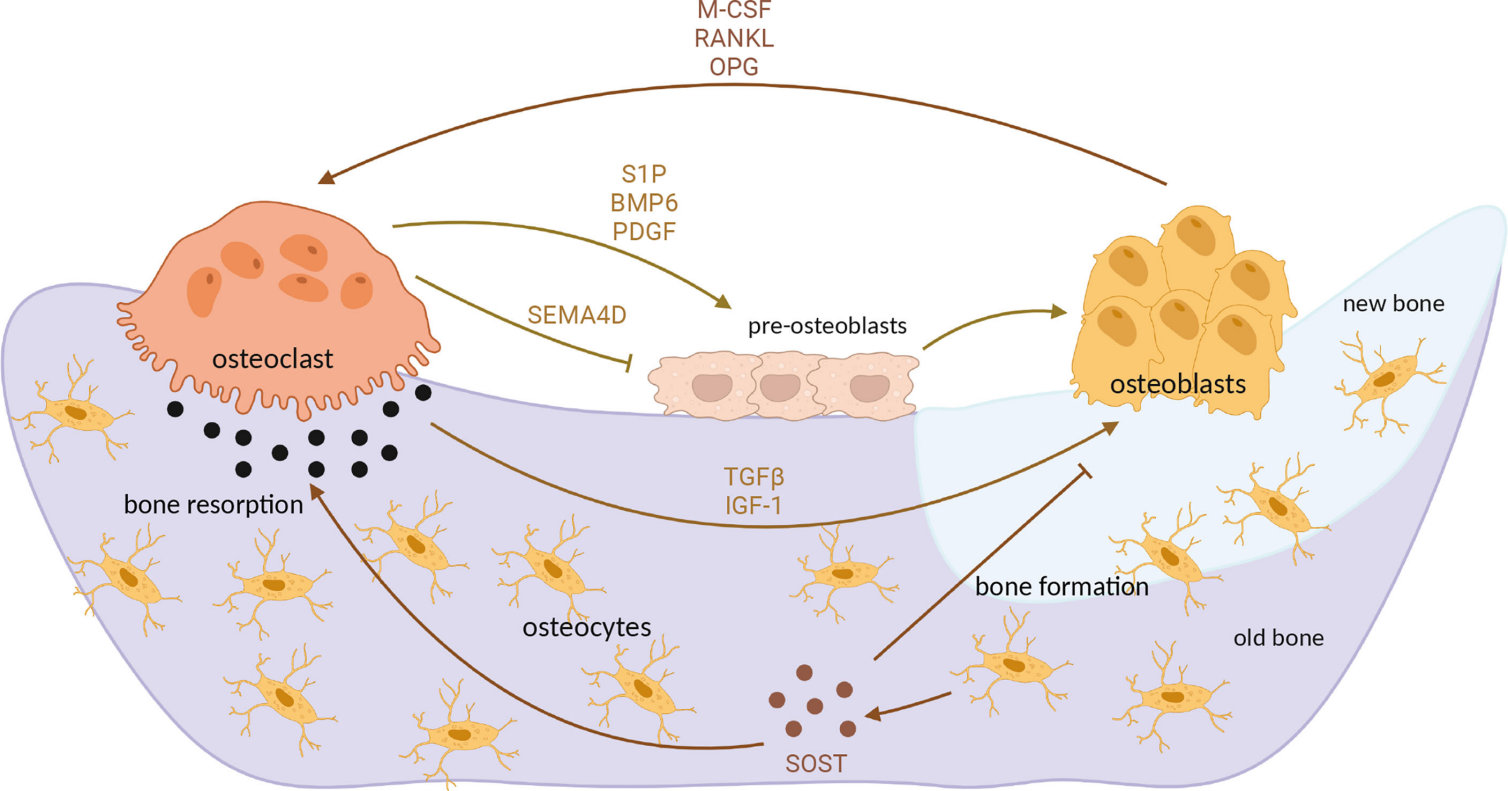
Interaction between osteoblasts and osteoclasts in bone remodeling process. Differentiated, mature osteoclasts secrete acids and matrix metalloproteinases which degrade mineralized bone. Bone resorption mediated by osteoclasts releases TGFβ and IGF-1 from bone matrix, which induce osteoblast activity and subsequent bone formation. M-CSF, RANKL and OPG secreted by osteoblasts additionally influence osteoclast differentiation and activity. In turn, osteoclasts secrete various factors which positively (S1P, PDGF) or negatively (SEMA4D) influence osteoblast differentiation. At the end of demineralization process, osteoblast precursors (preosteoblasts) are recruited at the resorption site, differentiating into mature osteoblasts which then form new, unmineralized matrix (osteoid). Upon mineralization, mature osteoblast differentiate into osteocytes which secrete sclerostin, additionally stimulating osteoclastogenesis but inhibiting osteoblast differentiation. OPG, osteoprotegerin; TGFβ, transforming growth factor β; IGF-1, insulin-like growth factor 1; S1P, sphingosine-1-phosphate; PDGF, platelet-derived growth factor; SEMA4D, semaphorin 4D; SOST, sclerostin. Image created by BioRender.com.

Differentiation of osteoclasts is under control of osteoblast paracrine factors, such as RANKL, interleukins (IL) 1 and 6 and Tumor necrosis factor α (TNFα) (50). Another important way of intercellular communication is direct cellular contact between osteoblasts and osteoclasts, driven mainly by Ephrin, Semaphorin 3A and FAS ligand-activated pathways (51). However, osteoclasts also in turn influence osteoblasts by secreting diffusible factors such as sphingosine-1-phosphate, Semaphorin 4D, platelet-derived growth factor and others, as well as by releasing growth factors from extracellular matrix (ECM) during bone resorption, in particular TGFβ1 and insulin-like growth factor (IGF-1) (50, 51). Additionally, osteocytes, cells derived from osteoblasts and embedded in bone matrix, secrete sclerostin (SOST), a protein which inhibits osteoblast differentiation but stimulates osteoclastogenesis (52) (**Figure 2**). TGFβ, a multifunctional cytokine, has been demonstrated to regulate osteoclastogenesis; however, its role in osteoclast maturation appears to be very complex, since TGFβ has both stimulatory and inhibitory effect on osteoclast precursors and mature osteoclasts (53–55), depending also on intracellular signaling pathways activated upon its binding to the cell surface (56).

## Role of Osteoclasts in Bone Remodeling

Bone remodeling process consists of several phases: 1) recruitment and activation of preosteoclasts and their differentiation into osteoclasts, 2) resorption of the mineralized matrix by mature osteoclasts through acidification of extracellular environment, 3) reversal - end of resorption process, apoptosis of osteoclasts and recruitment of preosteoblasts, and 4) deposition of osteoid by mature osteoblasts and subsequent mineralization (48, 57). Upon mineralization, mature osteoblasts undergo apoptosis or differentiate into quiescent osteocytes (58). The majority of new bone formation takes place on resorbed bone surfaces (59) and sites of bone remodeling activity are called basic multicellular units (BMUs), distributed throughout the skeleton in different stages of remodeling cycle, i.e. asynchronously (60).

Bone resorption is followed by bone formation in tightly controlled coupling process in order to preserve bone balance and prevent bone loss (59). As unique cell type which have capability to resorb mineralized bone matrix, osteoclasts have the crucial role in bone remodeling. The dissolution of bone mineral matrix (composed mainly of crystalline hydroxyapatite) is possible due to the osteoclast secretion of hydrochloric acid into resorption lacunae (61). However, besides their catabolic role in bone homeostasis, evidence from human diseases and mouse genetic models indicate that osteoclasts also have anabolic role in this process by coupling activity with osteoblasts (62, 63). Independently of their resorption activity, osteoclasts secrete coupling factors which most likely promote recruitment of osteoblast precursors to the bone surface (60). Osteogenesis-related mRNAs in osteoblasts (*Alph*, *RunX*2, *Col*1) are up-regulated in co-culture of osteoblasts and osteoclasts, indicating their mutual influence (64).

Among wide variety of potential coupling factors [presented in (60)], BMPs have significant role in this process, and central role might have BMP receptor type IA (65), as seen from studies on animal knockout models. Mice with deletion of BMPRIA in osteoclasts showed increased osteoblastic bone formation, which suggests that BMPRIA signaling in osteoclasts negatively regulates osteoblast differentiation and bone mass (66, 67). Another study demonstrated that BMPRIA deletion changed expression of several genes involved in osteoblast-osteoclast communication, notably *Cx43/Gja1* which encodes one of gap junction proteins (50). Recently, SMAD1/5 suggested to be regulatory pathway for osteoblast-osteoclast coupling *via* WNT and sphingosine kinase (SPHK1) (68). Collectively, BMPs act as important mediators in osteoblast-osteoclast communication and thus balance the rate of bone remodeling process (69), which could be of significant importance when considering potential therapies targeting BMP signaling pathways (65).

## BMPs and Osteoclasts – Models *In Vivo*

Better understanding of the important role of BMPs on skeletal development and bone homeostasis came from studies on genetically manipulated mice with global or conditional deletion of some of BMP ligands or their receptors (70, 71). *Bmp* genes and their downstream signal transducers are expressed early during development, before gastrulation (11). The critical role of BMP signaling during bone formation and developmental processes of whole body has been elucidated through numerous studies on genetically modified mice with conditional or global deletions of various Bmp genes. While some of global BMP deletions (BMP5, BMP6) have minor impact on development, complete loss of function of some BMPs results in prenatal (BMP2, BMP4) or early postnatal death (BMP7) (**Table 1**). To avoid embryonic lethality, for genetical studies on BMPs in mice a conditional knockout system (Cre-LoxP) has been used in further studies (80).

**Table 1:** An overview of BMP knockout rodent models.

Bmp ligand deleted	Type of deletion	Phenotype	Reference
BMP2	Global	Lethal - abnormal cardiac development	(72)
	Conditional (chondrocytes)	Chondrodysplasia	(73)
	Conditional (skeletally mature mice)	Trabecular bone loss	(74)
		Diminished osteoid formation	
		Impaired both osteoblast and osteoclast function	
BMP4	Global	Lethal - abnormal mesodermal differentiation	(75)
	Conditional (chondrocytes)	Minor effects on cartilage phenotype	(73)
BMP5	Nonsense mutation (naturally occurred)	Short ear phenotype and brachypodism due to the slowed formation of new cartilage	(76)
BMP6	Global	Minor sternal defects	(77)
BMP7	Global	Underdeveloped kidney mesenchyme, no eye development	(78)
		Skeletal patterning defects	(79)
		Lethal due to the kidney failure	

Among BMP knockout models listed in **Table 1**, BMP2 is most extensively studied. Conditional ablation of BMP2 in skeletally adult mice revealed that BMP2 affects functions in both osteoblasts and osteoclasts, with its deletion, in combination with deletion of BMP4, leading to the diminished osteoid formation and trabecular bone loss (74). Similar to BMP2 knockout, BMP4 knockout mice die before birth and show abnormal mesodermal differentiation (75). Interestingly, in contrast to BMP2 conditional deletion in chondrocytes, conditional deletion of BMP4 in these cells shows only minor changes in cartilage phenotype (73). During limb development in *Bmp2*, *Bmp4* and *Bmp7* conditional knockout mice, initiation of chondrogenesis and chondrogenic differentiation starts in the absence of both BMP2 and BMP4 or BMP2 and BMP7, however, both, BMP2 and BMP4 together are required for completion of osteogenesis (81). An opposite animal model, mice with overexpression of *Bmp*4 in osteoblasts developed osteopenia due to the increased osteoclastogenesis, implicating mutual influence between main bone cell types (82). It must be emphasized that BMP-induced ectopic bone formation does not mirror the real situation in bone microenvironment, since bone formation at ectopic site initially does not include osteoclasts, which are a significant factor not only in bone resorption, but also in new bone formation and its homeostasis (83).

Besides deletions of BMP ligands, models with mutations in BMP receptors were especially useful in studies of BMP signaling. Complete loss of BMP receptor type 1A due to the null mutation in Bmpr1A gene causes embryonic lethality and no mesoderm formation (84). However, conditional deletion of this gene targeted to osteoclasts caused increased bone volume and increased osteoblastic bone formation, indicating important role BMP signaling might have in osteoblast-osteoclast communication (67). Other transgenic mouse models involving BMP receptor genes are discussed in more detail in the subheading “BMP signaling “later in this review.

## BMP Function in Osteoclasts

It is known that BMPs coordinate many developmental processes, including body axis determination, germ layer specification and tissue morphogenesis (85), and that BMP signaling pathway remained conserved during evolution across distant animal species. In the cell, BMPs are produced as precursor proteins, consisting of a signal peptide, pro-domain and mature peptide. Upon cleavage of the signal peptide, precursor protein undergoes glycosylation and dimerization inside cytoplasm and is secreted in dimeric form as mature protein, whereas the pro-domain is cleaved (86). On the cell surface, BMPs bind to Type I or Type II BMP receptors which are transmembrane proteins with intracellular serine/threonine kinase domain. Activated receptors then mediate signal transduction mainly *via* canonical SMAD-dependent signaling pathway (SMAD 1/5/8 or SMAD 2/3) (87, 88).

The role of BMPs in bone formation is well described in literature (89). BMP2, -4, -5, -6, -7 and -9 exhibit high osteogenic activity (5, 34). It is known that BMP2 and -7 increase osteoblastic differentiation markers (34, 90), and that BMP signaling promotes chondrocyte differentiation (91). By acting on osteoblasts and chondrocytes, BMPs enable process of endochondral bone formation and ossification (92). In osteoblasts, BMPs act in a complex interaction with several signaling pathways, including Wnt, Notch, Hedgehog and FGF (89, 93). Loss of BMP function caused by genetic deletion of certain BMP genes and BMP receptors induces multiple skeletal defects in various mouse genetic models (80).

BMP activity in bone cells is additionally regulated by several proteins which act as BMP antagonists. The most important BMP inhibitors are noggin and chordin, which bind BMPs (especially BMP2 and -4) with high affinity, preventing thus their interaction with receptors (94). Addition of noggin to bone marrow cultures inhibited both osteoblast and osteoclast formation, whereas addition of noggin-specific antibody increased osteoblast progenitor formation (69). Hence, BMPs, in balance with noggin as their main antagonist, may provide baseline control for the bone remodeling rate (95).

Extracellular matrix is another important regulator of BMP biological activity in bone (96). Binding of TGFβ proteins to type IV collagen, a major component of ECM of basement membrane, has been demonstrated (97), as well as binding of BMP4 (98) and BMP7 (15). Besides collagen, other ECM components, such as small leucine-rich proteoglycans and fibrillins, can also bind BMPs and thus act as regulators of their bioavailability in the extracellular space (26, 34, 99). Extracellular BMP-binding components can act as its inhibitors by sequestering BMPs from their target cellular receptors, but can also promote BMP signaling by different mechanisms (100).

Although the role of BMPs in osteoblast maturation and function is well-known, their role in osteoclasts is not so extensively studied (92). Several studies demonstrated that osteoclasts endogenously express several BMP ligands (BMP2, BMP4, BMP6 and BMP7), BMP receptors (BMPR1A, BMPR1B and BMPR2) and SMAD proteins (57, 101–103). In particular, there are several studies underlining the role of BMP2 and BMP4 in osteoclastogenesis and bone resorption (82, 102–105). Transgenic mice overexpressing Noggin (inhibitor of BMP action) in osteoblasts showed decreased bone formation rate and significant decrease in osteoclast number, implicating the important role of BMP signaling in osteoclasts as well as in osteoblasts (82). Osteoclasts and osteoclast precursors express BMP receptors, which was confirmed in numerous studies (57, 67, 102, 103). These receptors are of key importance for intracellular BMP signal transduction, a process which enables BMPs to exert different effects on osteoclast maturation and function.

**Figure 3:**
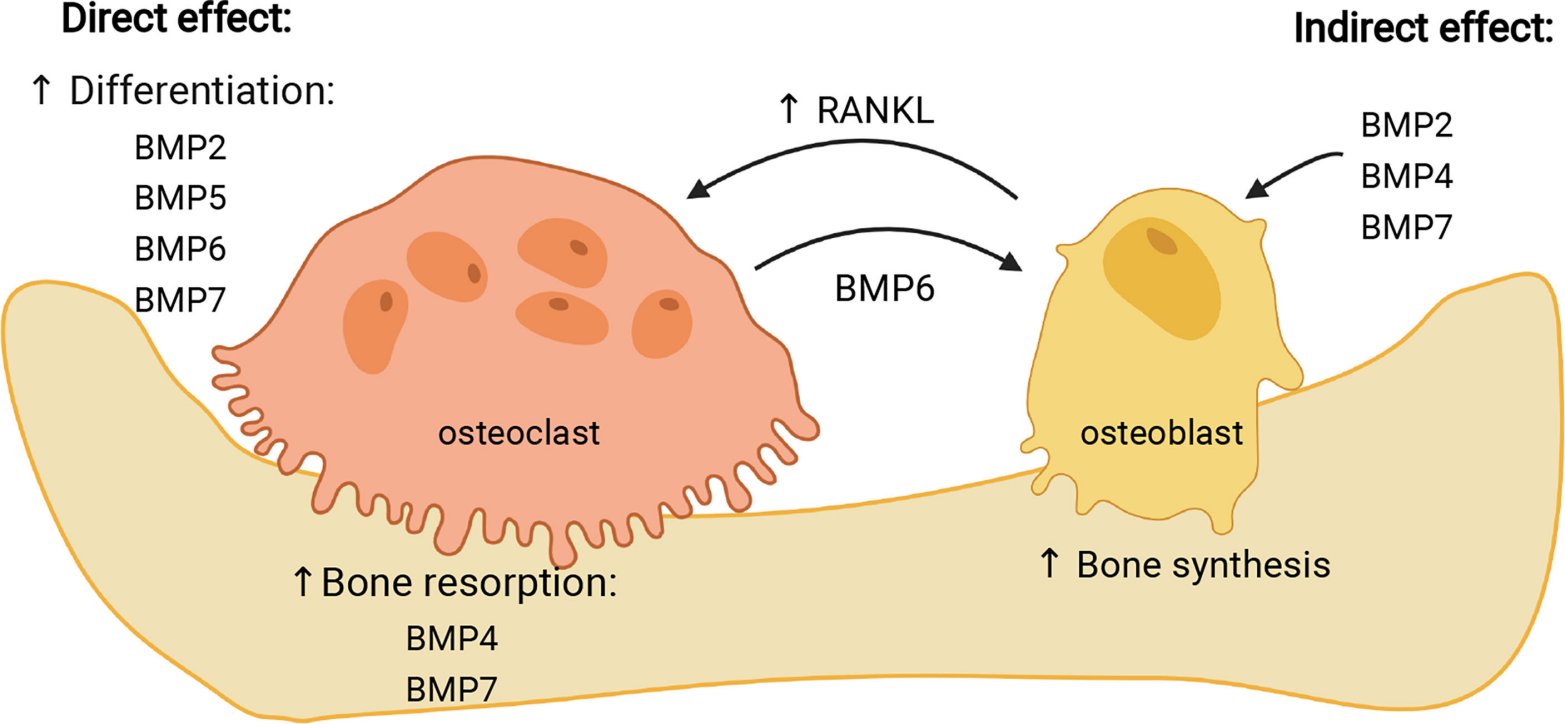
Effects of individual BMPs on osteoclasts. BMPs can influence osteoclasts either directly, stimulating their differentiation (BMP2, -5, -6, -7) and resorptive activity (BMP4, -7), or indirectly, through stimulation of osteoblasts (BMP2, -4, -7) which then increase expression of RANKL and stimulate osteoclast maturation. In addition, BMP6 expressed by osteoclasts stimulates osteogenic activity of osteoblasts. Image created by BioRender.com.

Various studies demonstrated different effects of particular BMPs on osteoclasts, depending on the model used and type of experimental cell treatment (**Table 2**). Most of studies performed so far report stimulatory effect of BMPs on osteoclast formation (117). In the following sub-section, the effect of most frequently studied BMPs on osteoclasts will be presented, which is also summarized in **Figure 3**.

**Table 2:** An overview of BMP action on osteoclasts.

BMP ligand	Effect on osteoclast activity	References
BMP2	Promotes osteoclast differentiation	(103)
	Stimulates osteoclasts in the presence of stromal cells	(106)
	Stimulates bone resorption in cultured osteoclasts	(104)
	Stimulates osteoclast formation in the presence of IL-1α	(107)
BMP4	Stimulates bone resorption by osteoclasts and promotes bone loss	(82)
		(104)
BMP5	Biphasic stimulatory effect on osteoclast generation, depending on concentration	(108)
		(109)
BMP6	Increases number of TRAP+ cells at optimal concentration, in higher concentrations its stimulatory effect declines	(109)
	Uncouples osteoblast from osteoclast activity, reduces bone resorption and increases bone formation in rat model	(110)
	BMP6 expression increased in mature osteoclasts, activated Wnt pathway to promote osteoblast differentiation and bone formation	(111)
BMP7	Increases osteoclast formation *in vitro* in combination with vitamin D3	(112)
		(69)
	Increases number of TRAP+ cells at optimal concentration	(109)
	Inhibits osteoclast differentiation in cultured C14+ monocytes	(113)
BMP9	Promotes osteoclast differentiation *in vitro*	(114)
	Increases bone resorption by mature osteoclasts in culture	(115)
	Inhibits osteoclastogenesis and bone resorption on *in vitro* and *in vivo* models	(116)

### Bmp2

BMP2, which has been the most studied of BMPs in osteoclasts as well as in osteoblasts, initially demonstrated stimulatory effect on osteoclast differentiation and activity only in the presence of stromal cells, which implicated indirect action of BMP2 on osteoclasts (106). Other studies *in vitro* also indicated the indirect effect of BMP2 on osteoclasts, acting *via* regulation of RANK expression in osteoblasts (118), and including 1,25(OH)_2_D_3_ as a mediator which decreases production of osteoprotegerin, accelerating thus osteoclastogenesis (119). However, a number of studies demonstrated that BMP2 derived from bone marrow macrophages has also a direct autocrine effect on osteoclast differentiation and maturation, activating canonical intracellular signaling pathway (69, 103, 105). It was also shown that BMP2 in osteoclasts can activate both canonical and non-canonical signaling pathway, depending on the stage of osteoclast differentiation, with p38 phosphorylation in the pre-fusion osteoclasts, and increased SMAD phosphorylation occurring at osteoclast fusion stage (102).

Recently, osteoblast-osteoclast contact *ex vivo* was facilitated by introduction of BMP2 immune complexes consisting of immobilized antibodies specific for BMP2 which sequestered endogenous BMP2. As a consequence, facilitated osteoblast-osteoclast interaction *in vitro* stimulated osteoblastogenesis and suppressed osteoclastogenesis, most probably *via* enhanced EphrinB2/EphB4 signaling pathway (120), suggesting the role of BMP2 not only in particular cell type, but also in their mutual communication.

### Bmp4

BMP4 is closely related to BMP2 and both molecules are required, not only for osteoblastogenesis, but also for proper osteoclastogenesis (69). Mouse overexpression models confirmed the stimulatory role of BMP4 in osteoclast differentiation (82, 121), most likely acting indirectly through stimulation of osteoblasts (69, 82). In bone marrow-derived stromal cells BMP4 was shown to induce expression of osteoprotegerin through the activation of p38 kinase, which could be the mechanism for regulation of osteoclast differentiation (122). However, BMP4, as well as BMP2, also acts directly on mature osteoclasts stimulating their bone-resorbing activity (104, 123).

*In vivo*, both BMP4 and BMP2 are essential for completion of osteogenesis (81). As seen from mouse knock-out models, BMP4 is also essential for mesoderm formation during development (75). Osteoclasts near the fracture site express BMP4, as well as BMP2 and BMP7, implicating their role in bone remodeling and fracture healing (124).

### Bmp5

In primary cultures murine bone marrow cells, BMP5 stimulated osteoclastogenesis, but with a biphasic effect, with higher concentrations (>300 ng/mL) being less stimulative on osteoclast formation than lower concentrations (0.1-100 ng/mL), and maximal effect was achieved at 1 ng/mL BMP5 (108). Similar study on primary rat bone marrow cells demonstrated stimulative effect of BMP5 on osteoclast-specific marker expression, it was significantly lower than for BMP2 or BMP4 (109). In cultured bone marrow cells, BMP5 likely acts by decreasing OPG and increasing RANKL mRNA expression, stimulating thus osteoclast differentiation (108). *In vivo*, numerous non-fatal skeletal defects were described in mice with inactivated *Bmp5* gene (76), suggesting the role of BMP5 in bone remodeling. The stimulatory effect of BMP5 seems to be more expressed in osteoblasts than in osteoclasts (108), and stimulation of osteoclastogenesis is enhanced in combination of BMP5 and BMP2 (125).

### Bmp6

Among multiple types of BMPs tested, BMP6 was one of most potent osteogenic BMPs (18, 109, 126), also due to its resistance to noggin (127), but it seems to be less potent in stimulation of osteoclasts than in osteoblasts (65, 108, 128). Structurally closely related to BMP5, BMP6 has also been shown to have stimulatory effect on osteoclasts at some concentrations, although both, BMP5 and BMP6, were less effective in stimulating osteoclastogenesis in comparison with BMP2 (108). BMP6 is expressed by mature osteoclasts and it was identified as one of the factors responsible for coupling bone resorption and osteoblast maturation, acting through increased activation of BMP pathways and activating thus osteoprogenitor cells (111). Osteoclasts contain relatively high levels of *Bmp6* mRNA, which could be important for regulation of overall bone homeostasis, not only for osteoblastic stimulation, but also for fine regulation of osteoclastogenesis (57). Systemic administration of BMP6 on rat model of osteoporosis *in vivo* increased osteoprotegerin serum levels uncoupling thus osteoclast from osteoblast activity (110). Interestingly, mice with inactivated BMP6 have only minor skeletal defects, such as prolonged ossification of sternum (77), but after revised phenotype, haemochromatosis with high iron content in organs has been discovered (129). Preferential stimulation of osteoblasts rather than osteoclasts gives advantage to BMP6 when considering its therapeutic use in bone healing (130, 131).

### Bmp7

Also known as osteogenic protein-1 (OP-1), BMP7 is, together with BMP2, one of BMPs with recognized therapeutic potential, first in animal studies and then in clinical trials (132). However, its osteogenic activity was accompanied by side effects including enhanced bone resorption and osteolysis at the osteotomy site (133, 134). Several studies *in vitro* showed that BMP7 promotes osteoclast formation in rodent bone marrow cell cultures, where its effect depends on the applied concentration (109, 112, 135). In combination with 1,25(OH)_2_D_3_, BMP7 stimulated not only osteoclast formation, but also resorption activity (112). In contrast, in human CD14+ monocyte culture, BMP7 inhibited osteoclast formation, apparently by down-regulation of transcription factor NFATc1, which is necessary for proper osteoclastogenesis (113). The reason for this difference could be in model of cell culture used (mouse or rat vs. human). It is possible that BMP7, similarly to the BMP2, acts on osteoclasts indirectly, through activation of osteoblasts (6).

### Bmp8

With its sequence being closely related to BMP7, BMP8, at first known as osteogenic protein 2 (OP-2) was identified in mouse embryos (16, 136). A recent transcriptomic analysis revealed that BMP8, similarly to BMP2,-4 and -7, can induce SMAD-signaling pathway in mesenchymal stem cells (137). BMP8 seems to have a protective role in osteoblasts exposed to glucocorticoids (138); however, studies about BMP8 action on osteoclasts are still lacking.

### Bmp9

First studies *in vitro* demonstrated positive effect of BMP9 on osteoclastogenesis. In human blood cord monocyte culture, BMP9 did not affect osteoclast formation, but increased their resorption activity, acting probably *via* SMAD1/5/8 and ERK1/2 pathway (115). Subsequent study on mouse spleen macrophages showed that BMP9 promoted proliferation and differentiation of osteoclast precursor cells in dose-dependent manner (114). However, a recently published study demonstrated an opposite efect of BMP9 on osteoclasts, suppressing RANKL-induced osteoclast differentiation of bone marrow macrophages *in vitro*, and preventing bone loss in mouse ovariectomy model *in vivo*, showing thus strong osteogenic effect (116).

## BMP Signaling in Osteoclasts

BMP stimulates the downstream signaling pathways by activating two types of BMP receptors. Type I and type II BMP receptors are the only known class of transmembrane cell surface receptors in humans with serine/threonine kinase activity. These two types of receptors share similar structural properties comprised of a relatively short extracellular domain and a single pass transmembrane protein with an intracelular serine/threonine kinase domain. Type II receptors are constitutively active, and after ligand binding they phospohorylate a Gly/Ser-rich domain of type I receptors and activate a kinase activity. Type I BMP receptors are Ser/Thr-protein kinase receptor R3 (ALK1), activin receptor type−1 (ACVR1/ALK2), BMP receptor type−1A (ALK3) and BMP receptor type−1B (ALK6), whereas BMP receptor type−2 (BMPR2), activin receptor type−2A (ACVR2A) and ACVR2B can function as type II BMP receptors (87, 139). Several studies revealed that osteoclasts express *Bmpr1a*, *Bmpr1b* and *Bmpr2* mRNA or protein (50, 67, 102). Regulation of BMP receptor expression in osteoclasts is not yet fully explained.

BMP ligands in osteoclasts act either through the canonical or non-canonical signaling pathways. The canonical pathway, also known as SMAD signaling pathway, involves three types of SMAD proteins: receptor SMADs (R-SMADs) transduce signals, common SMADs (Co-SMADs) support gene transcription activation and inhibitory SMADs negatively regulate BMP signaling. SMADs are homologues of *Drosophila melanogaster* Mad proteins (mothers against decapentaplegic) and *Caenorhabditis elegans* SMA proteins (small body size), and encode cytoplasmic proteins required for responsiveness to BMP superfamily ligands (140). Activated (phosphorylated) type I receptors recruit and phosphorylate pathway-specific R−SMADs (SMAD1, SMAD5 and SMAD8), which can form trimers with SMAD4 (Co-SMAD) and translocate to the nucleus where they target the genome *via* consensus SMAD-binding motifs, integrate with tissue-specific transcription factors and recruit chromatin remodeling machinery (141). A number of studies have shown that osteoclasts express SMADs as well as phosphorylated SMADs (68, 102, 103). An inhibition of SMAD signaling pathway leads to smaller and less active osteoclasts which suggests that BMP-mediated SMAD signaling plays a role in osteoclast fusion and activation (102, 135, 142). Deletion of *Smad4* in systems *in vitro* demonstrated that loss of *Smad4* during the early stages of osteoclast differentiation results in the loss of osteoclast differentiation as was measured by decreased expression of *Nfatc1* and *DC-STAMP*, as well as decrease in pSMAD2/3 expression (143). However, conditional deletion of *Smad4* in mature osteoclasts resulted in osteopenia due to increased osteoclast formation and bone resorption, and lead to an osteopenic phenotype caused by changes in the sensitivity to TGFβ signaling but not due to changes in BMP signaling (144). Additionaly, research on mice with conditionally deleted *Smad1/5* in osteoclast precursors led to mild bone gain due to reduced bone resorption and stimulated bone formation (68). Importance of canonical BMP signaling during the time of osteoclast fusion was shown when using dorsomorphin in fusion staged osteoclasts, where inhibition of type I receptors inhibited intracellular SMAD signaling and osteoclast differentiation (102, 103, 143).

Studies on osteoclasts and osteoclast precursors in transgenic mouse models have indicated different roles of type I and type II BMP receptors in osteoclast formation and bone resorption with complex mechanisms of signal transduction involving both canonical and non-canonical pathways. Conditional knockout of the BMPRIa receptor in osteoclast progenitors resulted in a decrease in osteoclastogenesis and expression of *DC-STAMP* (145), the master regulator required for preosteoclast fusion (44, 146). Its expression is regulated by an essential transcription factor, Nfatc1, which is activated downstream of RANKL and BMP-signaling pathway (147). Inhibition of BMP signaling leads to a decrease in *DC-STAMP* and *Nfatc1* gene expression, resulting in fewer, smaller, and less active osteoclasts, showing the requirement of BMP signaling in preosteoclast fusion (82, 103). In osteoclasts derived from BMPRIA conditional knockout mice mRNA levels of *Pu.1* and *Mitf*, transcription factors required for osteoclast commitment and early differentiation, were increased, whereas the mRNA levels of late osteoclast differentiation markers and *Nfatc1* were downregulated, indicating that BMPRIA deficiency enhanced the initial differentiation but disrupted the maturation of osteoclasts (145). Addition of (soluble) BMPRIA on osteoclast formation in bone marrow macrophage cultures suppressed osteoclast formation induced not only by the combination of RANKL and BMP2, but also by RANKL alone (105). Therefore, BMP signaling may be required for RANKL-mediated osteoclastogenesis.

In contrast to the deletion of *Bmpr1a*, in mice with global knockout of *Bmpr1b* enhanced proliferation and survival of osteoclast precursor was observed, along with reduced apoptosis and reduced resorption activity (148). Despite decreased resorption, these mice showed transient osteopenia, probably due to the compromised differentiation of osteoblasts where BMP signaling also plays an important role. However, osteoblast and osteoclast activity *in vivo* were not observed (148), implicating more subtle role of BMPRIB receptor in the regulation of bone remodeling.

Mutations in BMPRII are more extensively explored in diseases not related to the bone metabolism, such as pulmonary arterial hypertension (PAH) (149). A study with bone marrow-derived *Bmpr2*-deficient osteoclasts showed decreased osteoclast differentiation and resorptive activity (102). Mice with *Bmpr2* conditional knockout had increased bone volume and trabeculae with osteopetrotic phenotype due to the reduced bone resorption. At the cellular level, these mice had changes in the non-canonical signaling (MAPK) and no changes in the canonical signaling (SMAD) pathway, as was measured by levels of phosphorylated and nonphosphorylated forms of SMAD proteins and downstream elements of non-canonical signaling pathway, suggesting rather complex mechanism of intracellular signalization with non-canonical pathway being important for proper osteoclastogenesis (102).

The non-canonical BMP signaling pathway consists of mitogen-activated protein kinase (MAPK) and several downstream signaling molecules, including c-Jun N-terminal kinase (JNK), mitogen-activated protein kinase 38 alpha (p38α) and extracellular regulated kinases (ERK), all of which are activated by BMP 2 in osteoclasts (102, 115). One of upstream signaling molecules of the non-canonical signaling pathway, TGFβ Activated Kinase 1 (TAK1), is required for osteoclast differentiation, as seen from the specific knockout of TAK1 in osteoclasts, which lead to an osteopetrosis-like phenotype with decreased resorptive activity (150). TAB1, an activator of TAK1 protein, participates together with TAK1 in the BMP signaling pathway. It was found that another regulatory molecule, X-linked inhibitor of apoptosis protein (XIAP), serves as an adaptor protein linking the BMP receptors and TAB1-TAK1 complex. XIAP was determined as a TAB1-binding protein and interacts not only with TAB1 but also with BMP type I and type II heteromeric receptor complex, linking BMP signaling pathway with intracellular regulators of osteoclastogenesis (151).

**Figure 4:**
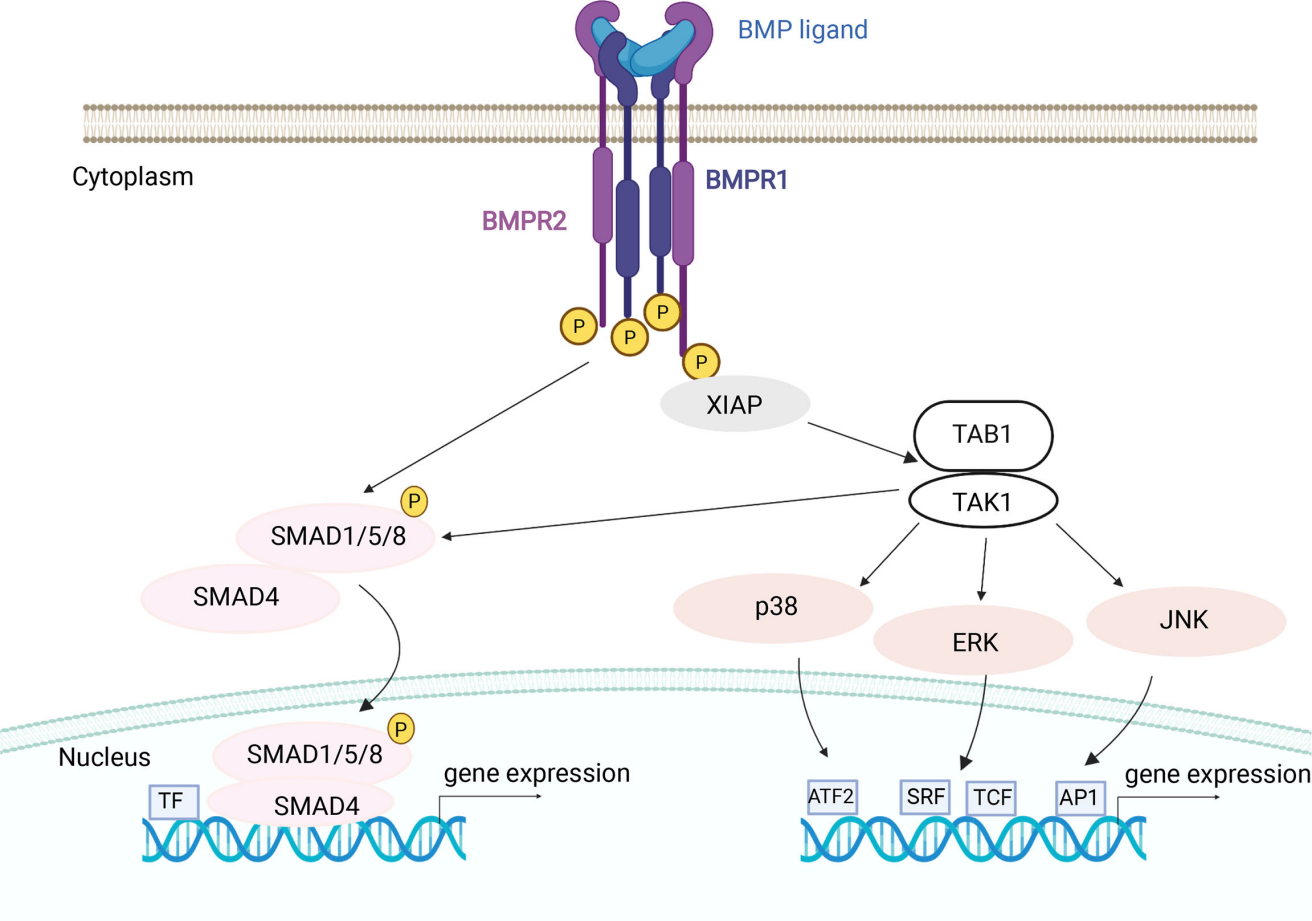
BMP mechanisms of canonical and non-canonical signaling in osteoclasts. After BMP ligand binding type II receptors phosphorylate (P) the type I receptors. Activated type I receptors recruit and phosphorylate canonical pathway specific R-SMADs (SMAD1/5/8) which, with the help of co-SMAD (SMAD4), transduce the signal into the nucleus. The non-canonical BMP signaling pathway is transduced by the recruitment of TAB1/TAK1 complex through XIAP. Activated TAK1 kinase can stimulate the downstream non-canonical MAP kinase effector proteins or canonical SMAD proteins. Activated MAPKs can translocate to the nucleus to phosphorylate a number of transcription factors (TF), thereby changing target gene transcription. BMPR1 and BMPR2, BMP receptor 1 and 2; XIAP, X-linked inhibitor of apoptosis protein; TAK1, TGFβ Activated Kinase 1; TAB1, TGFβ Activated Kinase 1 binding protein; p38, mitogen-activated protein kinase 38; JNK, c-Jun N-terminal kinase; ERK, extracellular regulated kinases; SRF, serum response factor; TCF, ternary complex factor family member; AP1, activator protein 1 complexes; ATF2, activating transcription factor 2. Image created by BioRender.com.

Besides BMP2, which activates MAPKs, ERK1/2, JNK and p38 in osteoclasts (102), it has been shown that BMP9 in osteoclasts also stimulates the activation of two signaling pathways, as seen from activation of both SMAD1/5/8 and ERK1/2 (115), suggesting that BMPs can activate both non-canonical and canonical signaling and that TAK1 has a crucial role in this process (115, 151, 152) (**Figure 4**).

## BMPs and Bone Loss in Inflammatory Conditions

In bone healing, inflammatory response, a sequentional process involving complex interaction between multiple cell types, modulates microenvironment at the fracture site, and is crucial in initial phase of fracture healing (153). The significance of inflammatory process in fracture healing is additionally underlined by studies on animal models where healing is delayed in the absence of proinflammatory cytokines such as interleukin-6 (IL-6) or tumor necrosis factor-α (TNFα) (154, 155). During fracture healing, mesenchymal stem cells are recruited from the periosteum and bone marrow and differentiate into chondrocytes and osteoblasts which form callus and new bone (156). Among numerous cytokines and growth factors involved in this process, BMPs released by immune and osteoprogenitor cells play a crucial role in inducing osteogenic differentiation following inflammatory phase. Studies on inflammatory and autoimmune disorders pointed to the fine regulation of this process and to the complex interaction between BMPs and inflammatory cytokines. Mostly studied conditions are rheumatoid arthritis (RA) and ankylosing spondylitis (AS), where BMPs seem to have opposite roles. In RA, increased expression of BMP2, -6 and -7 observed in synovial fluid of RA patients indicated the role of BMPs in the development of this disease, probably by inducing proinflammatory phenotype of endothelial cells, stimulating adhesion of monocytes to the endothelium and, additionally, osteoclast differentiation, which subsequently leads to the bone loss observed in RA patients (157). On the other hand, enhanced BMP signaling in AS stimulates osteogenesis and induces heterotopic endochondral bone formation (158, 159). Proinflammatory cytokines upregulate expression of BMP2 and -6, indicating association between BMP activity and inflammatory processes in affected joints (160).

Since BMPs belong to the TGFβ superfamily of proteins, it is noteworthy that patients with chronic inflammation have elevated serum TGFβ levels, due to macrophage activation at the inflammation site. In those patients, decreased bone density is often observed, most likely due to the enhanced osteoclastic bone resorption stimulated by TGFβ (161). In parallel, chronic exposure of osteoblasts to TGFβ leads to the loss of their functionality, probably due to the continuous SMAD2/3 activity with concomitant decreasing of SMAD1/5/8 activity, which is otherwise activated by BMPs (162). Similarly, bone loss observed in affected joints of patients with RA is attributed to the increased osteoclastogenesis enhanced by proinflammatory cytokines (IL-1, IL-6, IL-17, TNFα), which induce RANKL expression in stromal cells and thus stimulate osteoclast precursors (163). IL-1 also directly stimulates osteoclast activity, even in absence of stromal cells (164). Proinflammatory cytokines not only stimulate osteoclastogenesis, but also inhibit osteoblast differentiation contributing to the overall bone loss (158). For example, TNFα acts opposite to the osteogenic transcriptional factors induced by BMPs (165), whereas IL-1 inhibits recruitment and migration of osteoblasts (166). Therefore, in addition to regulation of bone remodeling, BMPs are also involved in inflammatory conditions which indirectly affect bone homeostasis (167).

## Bone Resorption in Clinical Use of BMPs 

In order to overcome complications associated with non-healing bone fractures, therapeutic concepts using BMPs were developed (83). Numerous studies confirmed the effectiveness of BMPs in promoting osteogenesis (18, 168). In clinical use, therapeutic BMP devices usually consist of bovine collagen matrix as a carrier with added BMP2 (Infuse Bone Graft) or BMP7 (Osigraft) as an active substance (132, 169). However, complexity of biological effects of BMPs lead to side effects *in vivo* which were not observed in experimental systems *in vitro*, among which most prominent effect was stimulation of osteoclastic bone resorption (117). First clinical studies involving BMP-based therapies, namely BMP2, resulted in increased bone resorption rather than formation (170, 171), suggesting the role BMPs have in osteoclast activation (172) and subsequent osteolysis (142). When applied to the bone, BMP2 and BMP7 caused increased bone resorption, as demonstrated in preclinical (173) as well as in clinical studies (174, 175). Although it is well known that BMPs induce new bone formation, conditional deletion of BMP signaling in osteoblast appeared to have an inhibitory effect on osteoclastogenesis, implicating the complex role BMPs have on bone remodeling *in vivo* (176). Additionally, mice overexpressing BMP4 developed osteopenia due to the increased osteoclast number (82). Based on *in vitro* and *in vivo* studies, it became obvious that effect of BMPs on bone *in vivo* is a result of a stimulation of not only osteoblasts, but also osteoclasts and their progenitors which express BMP receptors (104, 177). It was also shown that BMP2 stimulates osteoclast formation in the presence of proinflammatory cytokine IL-1α and therefore it could enhance bone resorption due to the inflammatory environment at the site of the surgery (107). Furthermore, in ectopic bone formation the development of osteoclasts induced by BMP2 was demonstrated (178).

For BMP2 and BMP7, which were first BMPs tested in clinical trials, it was shown that, although promoting osteoblast differentiation, have significant impact also on osteoclasts, resulting in a net bone loss (83). Indeed, in clinical studies using BMP7 for distal radial osteotomy (133) and spinal fusion surgery (175, 179), pronounced bone resorption was observed. This effect was especially prominent in patients receiving BMPs in spinal fusion (95, 170, 180), whereas use of BMP7 in unstable thoracolumbar fracture resulted in segmental collapse due to the severe bone resorption (134). This pronounced effect on vertebrae was likely caused by the stimulatory effect of large amounts of BMP2 and BMP7 used on osteoclasts at endosteal and trabecular surfaces (83). The stimulation of endosteal osteoclasts could be an important step in bone healing, removing nonfunctional bone pieces following a fracture, accompanied by parallel formation of the new bone tissue (173), however, in clinical use, this BMP-induced osteoclast stimulation could lead to unwanted osteolysis. Further, if applied BMPs promote both bone resorption and formation, the end result may be impaired healing (181). The effect of BMP2 and BMP7 in clinical trials depends also on the amounts used; however, large amounts of BMPs usually used in spinal fusion may lead to the increased resorption in localized areas (95). Also, in currently available commercial devices, large amounts of BMP2 or -7 significantly exceed the biological need. Increased bioavailability due to the large amounts of applied BMP2 or -7 could finally result in unwanted side effects (174, 175), although studies on animal models demonstrated that systemic BMP2 and -7 will not stimulate generalized bone loss (182).

Recently, a new osteogenic device was developed, using BMP6 as an active substance and autologous carrier made from the peripheral blood (83, 183). This device, named Osteogrow, could overcome the limits of previously used BMP2 and BMP7 which were applied in large concentrations (131). BMP6 uses most of BMP receptors type I for signal transduction and stimulates osteoblast activity in cell cultures (102, 148). Studies of osteoclast cultures *in vitro* demonstrated preferential expression of *Bmp6* mRNA compared to expression of other BMPs (57); however, osteoclastogenesis *in vitro* is more stimulated by addition of BMP2 or BMP5 than by BMP6 (108). Although data on specific BMP6 effects on osteoclasts are scarce, it seems that BMP6 stimulates osteoblasts more than osteoclasts, which puts this protein into advantageous position regarding potential clinical use when compared to other BMPs. Indeed, in recently published clinical studies which applied Osteogrow in patients with distal radius fracture and high tibial osteotomy, no side effects related to the use of BMP6 were recorded, and no osteolysis or other signs of increased osteoclast activity were observed in these patients (184, 185). Therefore, when considering BMPs as therapeutics in delayed bone healing and other complex orthopaedic indications, the potential effect of particular BMPs on osteoclast proliferation and activity would be of great clinical importance.

## Concluding Remarks

A large number of *in vitro* and *in vivo* studies indicate that osteoclasts are not merely bone-degrading cells, but they also have an important function in osteoblast activation, bone remodeling and maintenance of bone homeostasis. Among numerous signaling molecules regulating their differentiation and activity, BMPs in particular seem to have important role in regulating osteoblast-osteoclast communication. Since BMPs have a therapeutic potential as bone-healing agents, it is of major importance to consider their effects on osteoclasts as well as on osteoblasts, in order to avoid potential unwanted side effects such as increased bone resorption and osteolysis.

## Author Contributions

TB-N and VK wrote draft of the manuscript and created figures, SV designed review content, edited first draft and approved final version. All authors contributed to the article and approved the submitted version.

## Funding

This article was supported by the Scientific Center for Excellence for Reproductive and Regenerative Medicine (project “Reproductive and regenerative medicine – exploration of new platforms and potentials” GA KK.01.1.1.01.0008 funded by the EU through the ERDF).

## Conflict of Interest

The authors declare that the research was conducted in the absence of any commercial or financial relationships that could be construed as a potential conflict of interest.

## Publisher’s Note

All claims expressed in this article are solely those of the authors and do not necessarily represent those of their affiliated organizations, or those of the publisher, the editors and the reviewers. Any product that may be evaluated in this article, or claim that may be made by its manufacturer, is not guaranteed or endorsed by the publisher.
